# Preparation of a Defined Gluten Hydrolysate for Diagnosis and Clinical Investigations of Wheat Hypersensitivities

**DOI:** 10.3390/nu10101411

**Published:** 2018-10-02

**Authors:** Herbert Wieser, Katharina A. Scherf

**Affiliations:** Leibniz-Institute for Food Systems Biology at the Technical University of Munich, 85354 Freising, Germany; h.wieser2@gmx.de

**Keywords:** celiac disease, diagnosis, gliadin, gluten, glutenin, non-celiac gluten sensitivity, oral food challenge, pepsin, wheat allergy

## Abstract

Gluten is the trigger for celiac disease (CD), non-celiac gluten/wheat sensitivity (NCGS), and wheat allergy. An oral food challenge is often needed for diagnosis, but there are no standardized gluten challenge materials with known composition available. To fill this gap, two materials, commercially available gluten and a food-grade gluten hydrolysate (pepgluten), were extensively characterized. Pepgluten was prepared from gluten by incubation with a pepsin dietary supplement and acetic acid at 37 °C for 120 min. The components of pepgluten were crude protein (707 mg/g), starch (104 mg/g), water (59 mg/g), fat (47 mg/g), dietary fiber (41 mg/g) and ash (11 mg/g). The protein/peptide fraction of pepgluten (1 g) contained equivalents derived from 369 mg gliadins and 196 mg glutenins, resulting in 565 mg total gluten equivalents, 25 mg albumins/globulins, 22 mg α-amylase/trypsin inhibitors and 48 mg pepsin capsule proteins. The slightly acidic, dough-like smell and bitter taste of pepgluten could be completely camouflaged in multivitamin juice with bitter lemon, grapefruit juice, or vegetable and fruit smoothies. Thus, pepgluten met the criteria for placebo-controlled challenges (active and placebo materials are identical regarding appearance, taste, smell, and texture) and is appropriate as a standard preparation for the oral food challenge and clinical investigations to study wheat hypersensitivities.

## 1. Introduction

Wheat hypersensitivities can be classified into celiac disease (CD) and related disorders (dermatitis herpetiformis Duhring and gluten ataxia), non-celiac gluten/wheat sensitivity (NCGS), non-IgE-mediated allergies and IgE-mediated allergies such as food allergy, skin allergy, respiratory allergy (e.g., baker’s asthma), and wheat-dependent exercise-induced anaphylaxis (WDEIA) [1,2]. Additionally, a subgroup of individuals suffering from irritable bowel syndrome (IBS), especially diarrhea-predominant IBS, appears to be sensitive to wheat products and may profit from adhering to a gluten-free diet [3,4].

A wide variety of wheat proteins may trigger these hypersensitivities in susceptible individuals with a certain genetic predisposition (HLA-DQ2/8-positive) or sensitization. The causative factors in wheat are gluten (gliadins and glutenins, 70–80% of wheat proteins) and non-gluten proteins (albumins/globulins, 20–30% of wheat proteins). Classically, wheat proteins are subdivided according to extractability into albumins/globulins soluble in aqueous salt solution: gliadins are soluble in aqueous alcohols, and glutenins soluble in aqueous alcohols only after reduction of disulfide bonds [5]. Gluten comprises more than one hundred single proteins and serves as a source of nitrogen and amino acids for the wheat germ, whereas albumins/globulins mainly contain metabolic and protective proteins such as enzymes and enzyme inhibitors. Gluten proteins are hydrophobic, compact, and characterized by repetitive amino acid sequences rich in glutamine and proline, which makes them resistant to human gastrointestinal enzymes [6]. Based on amino acid sequence similarities, gliadins are subdivided into the gluten protein types ω5-, ω1,2-, α- and γ-gliadins and glutenins into glutenin-bound ωb-gliadins, high-molecular-weight (HMW) and low-molecular-weight (LMW) glutenin subunits (GS) [7]. While CD is caused by gluten only, wheat allergy occurs following sensitization with both gluten and non-gluten proteins. In the case of NCGS, α-amylase/trypsin-inhibitors (ATIs) belonging to non-gluten proteins have been identified as activators of innate immunity and adjuvant of several inflammatory reactions [8,9], but gluten proteins may be involved as well.

The differential diagnosis of wheat hypersensitivities is complex and requires a high level of clinical suspicion. Especially in inconclusive cases, open or single- or double-blinded oral challenge of the patients with the suspected triggering factor needs to be performed. A 14-day provocation test with gluten is necessary to achieve a clear diagnosis in CD patients who have already voluntarily adopted a gluten-free diet [10]. Stepwise challenges with half-logarithmic dose increments of wheat proteins at intervals of at least 20 min are needed to diagnose an immediate wheat allergy [11,12]. For WDEIA, gluten ingestion is combined with exercise or other cofactors [13]. The diagnosis of NCGS and diarrhea-predominant IBS is known to be especially difficult due to the lack of specific biomarkers and frequent placebo or nocebo effects [2,14,15]. Therefore, a double-blind, placebo-controlled food challenge is regarded as the gold standard to establish whether wheat is involved in symptom induction [16], although this is difficult to undertake in daily clinical practice [3]. 

A number of different materials, for example, wheat bread [17] or cookies [18], udon noodles [19], wheat-containing daily meals [4], and wheat gluten as is [13], as gastrosoluble capsules [16,20] or added to bakery products such as muffins [21] have been used for oral challenge. However, the amount and composition of wheat proteins used in these materials were often not determined or indicated. For example, commercial wheat gluten (vital gluten) has been frequently assumed to consist of 100% gluten proteins, but the protein content is typically only about 70–80% based on fresh weight [21]. The gluten-containing foods used for the oral challenge should be indistinguishable from the gluten-free placebo in taste, smell, texture, and appearance, and contain comparable amounts of carbohydrates, dietary fiber, fat, and protein. The challenge food should provide adequate immunoreactive protein in a reasonable portion size, be easily applicable, closely replicate the usual form of the food, and its qualitative and quantitative composition should be known [2,11]. A dose of 8 g of gluten with a defined ATI content (at least 3 g ATIs per 100 g of gluten) is recommended for diagnosis of NCGS [2]. Already in 2004, the European Academy of Allergy and Clinical Immunology demanded that challenge tests should be carried out with standardized foods guaranteeing the safety of patients, practical feasibility, and the comparability of results between different study centers [22]. Despite several meetings of expert groups, no standardized preparation has so far been developed for the diagnosis of wheat hypersensitivities, although there is a clear need, especially for NCGS where the diagnosis still relies on exclusion of all other possible causes as long as specific biomarkers for NCGS have not been identified. Therefore, the aim of this study was to thoroughly characterize wheat gluten and prepare a well-defined wheat gluten hydrolysate (called “pepgluten” in the following), which fulfills the recommendations stated above and is suited for oral food challenge in clinical and scientific studies on wheat hypersensitivities.

## 2. Materials and Methods 

### 2.1. Reagents and Materials

All reagents and chemicals were purchased from Merck (Darmstadt, Germany), Sigma-Aldrich (Steinheim, Germany), AppliChem (Darmstadt, Germany), or Serva (Heidelberg, Germany) in analytical grade or higher. Water for reversed-phase high-performance liquid chromatography (RP-HPLC) was purified with an Arium 611VF water purification system (Sartorius, Goettingen, Germany). Prolamin Working Group (PWG)-gliadin [23] used for calibration was kindly provided by Prof. Dr. Peter Koehler, chairman of the PWG. Wheat gluten “vital” (batch no. 1607220051) was obtained from Hermann Kröner GmbH (Ibbenbueren, Germany) and contained ≤8% moisture, ≤0.9% ash and ≥78% protein (nitrogen × 6.25, based on fresh weight) according to the manufacturer’s product specification sheet (called “gluten” in the following). Pepsin capsules for use as a dietary supplement (153 mg pepsin, corresponding to 3 × 10^6^ albumin digestion units per capsule) were purchased from Dr. Clark Store (Chula Vista, CA, USA). Sodium hydroxide (“baker’s brine”) was provided by Minerva (Calbitz, Germany). Vinegar essence (Surig^®^, Speyer & Grund, Meerane, Germany, 25% acetic acid), mineral water (Adelholzener, Siegsdorf, Germany, in a glass bottle) and different fruit juices and smoothies were obtained from a local supermarket. According to the label, the mineral water contained 508 mg/L salts (nitrate < 0.3 mg/L). Acetic acid (pH 3.0) was prepared by mixing 900 mL mineral water and 100 mL vinegar essence.

### 2.2. Standard Analyses

The moisture and ash contents were determined according to International Association for Cereal Science and Technology (ICC) Standard Methods 110/1 and 104/1, respectively. The crude protein content (nitrogen × 5.7 for wheat) was analyzed according to ICC Standard Method 167 using a TruSpec Nitrogen Analyzer (Leco, Kirchheim, Germany) calibrated with ethylenediamine- tetraacetic acid (EDTA). The content of starch was determined according to AOAC Official Method 996.11 using the Total Starch Assay Kit (Megazyme, Bray, Ireland) [24]. The analysis of total dietary fiber was carried out with the Total Dietary Fiber Assay Kit (Sigma-Aldrich, Steinheim, Germany) based on a combination of enzymatic and gravimetric methods. The fat content was determined gravimetrically after extraction in hydrochloric acid (25%, *v*/*v*) and addition of toluene [25]. All analyses were done in triplicates each for gluten and pepgluten.

### 2.3. Contents of Albumins/Globulins, Gliadins and Glutenins in Gluten

The extraction of modified Osborne fractions was adapted from Wieser et al. [26] as follows: Gluten (20.0 mg, *n* = 3) was first extracted with 3 × 1.5 mL of salt solution (0.4 mol/L NaCl with 0.067 mol/L Na_2_HPO_4_/KH_2_PO_4_, pH 7.6, at 20–22 °C) to obtain albumins and globulins (ALGL). In a separate experiment, gluten (20.0 mg) was stepwise extracted with 3 × 1.5 mL 60% (*v*/*v*) ethanol to obtain a mixture of ALGL and gliadins (GLIA) and then with 3 × 1.5 mL glutenin extraction solvent (50% (*v*/*v*) 1-propanol and 0.05 mol/L Tris-HCl, pH 7.5, with 2 mol/L (*w*/*v*) urea and 0.06 mol/L (*w*/*v*) dithiothreitol (DTT)) at 60 °C under nitrogen to obtain glutenin subunits (GLUT). Each extraction step was performed by vortex mixing for 2 min and magnetic stirring for 30 min followed by centrifugation (3750× *g*, 25 min, 22 °C). The corresponding three supernatants were combined, made up to 5.0 mL with the respective solvent and filtered (0.45 µm). 

### 2.4. RP-HPLC for Gluten Proteins

The quantitation of protein fractions was performed by RP-HPLC on a Jasco XLC instrument (Jasco, Gross-Umstadt, Germany) with a C_18_ column (Acclaim^TM^ 300, 2.1 × 150 mm, 3 µm, 30 nm, Thermo Fisher Scientific, Braunschweig, Germany) at 60 °C. Elution solvents were 0.1% (*v*/*v*) trifluoroacetic acid (TFA) in water (A) and 0.1% (*v*/*v*) TFA in acetonitrile (B) at a flow rate of 0.2 mL/min with the following gradient: 0 min 0% B, 0.5 min 24% B, 20 min 56% B, 20.1–24.1 min 90% B, 24.2–30 min 0% B. The injection volumes were 20 µL (ALGL, GLUT) and 10 µL (ALGL + GLIA) and protein absorbance was detected at 210 nm. PWG-gliadin [23] dissolved in 60% (*v*/*v*) ethanol was used for external calibration. The contents of ωb-gliadins, HMW-GS and LMW-GS were calculated relative to the total area of GLUT as described earlier [27]. To obtain the GLIA content, the chromatogram of the ALGL fraction was subtracted from that of the ALGL + GLIA fraction using the Chrompass software (version 1.2, Jasco, Gross-Umstadt, Germany). Then, the contents of ω5-, ω1,2-, α- and γ-gliadins were calculated from the absorbance area of each type relative to the total area.

### 2.5. Optimization of Gluten Hydrolysis

Different ratios of enzyme (E), gluten (G) and solvent (S) were tested. The first assay was performed according to Dorum et al. [28] (E:G:S = 1:25:1667). Gluten (3.8 g) was suspended in 260 mL HCl (0.01 mol/L, pH 2.0, 37 °C) under magnetic stirring, 153 mg pepsin (1 capsule) were added and stirred for 120 min at 37 °C. The next assays were performed with acetic acid as solvent prepared by mixing mineral water with acetic acid essence until pH 3.0 was reached. To reduce the ratio of solvent, different incubations (120 min at 37 °C each) were done using E:G:S ratios of 1:25:200, 1:25:150 and 1:25:100. Aliquots (0.5 mL) were taken from all assays for analysis before addition of the enzyme (0 min) and after 10, 20, 40, 60, 90, and 120 min and heated for 10 min at 95 °C to inactivate pepsin. The remaining portions of the hydrolysates obtained after 120 min were lyophilized. In order to reduce the amount of enzyme, different incubations at 37 °C were performed using E:G:S ratios of 1:50:200, 1:100:400 and 1:200:800. After 120 min, aliquots (0.5 mL) were taken and inactivated as described above. 

### 2.6. Preparation of Pepgluten

Twenty-five capsules (3.8 g pepsin) were dissolved in 380 mL acetic acid (pH 3.0) at 37 °C. Gluten (95 g) was added (E:G:S = 1:25:100) and magnetically stirred for 120 min at 37 °C. The assay was cooled, frozen and lyophilized. The dried digest was mixed with boiling mineral water (200 mL) and heated for 10 min, cooled, frozen and lyophilized. The dried material was milled (A10, IKA-Werke, Staufen, Germany) and stored in a vacuum desiccator over baker’s brine until a constant weight was reached.

### 2.7. RP-HPLC for the Gluten Hydrolysates

The 0.5 mL aliquots (see Section 2.5) were diluted 1:20 with 0.1% (*v*/*v*) TFA, filtered (0.45 µm) and 50 µL of the filtrates were analyzed on a Thermo Finnigan Spectra System (Thermo Fisher Scientific) with ChromQuest software using an Aeris Peptide XB-C_18_ column (2.1 × 150 mm, 3.6 µm, Phenomenex, Aschaffenburg, Germany). Elution solvents were 0.1% (*v*/*v*) TFA in water (A) and 0.1% (*v*/*v*) TFA in acetonitrile (B) at a flow rate of 0.2 mL/min at 22 °C and a detection wavelength of 210 nm with the following linear gradient: 0 min 0% B, 5–60 min, 0–40% B, 60.5–66.5 min 90% B, 70–76 min 0% B.

### 2.8. Sodium Dodecyl Sulfate-Polyacrylamide Gel Electrophoresis (SDS-PAGE)

Gluten and gluten hydrolysates were characterized by SDS-PAGE on a homogeneous NuPAGE 10% polyacrylamide-Bis-Tris gel (10 × 1 mm wells Invitrogen, Carlsbad, CA, USA) [29,30]. A mixture of proteins was used as size standard (PageRuler^TM^ Unstained Protein Ladder, Thermo Fisher Scientific). Gluten samples (4 mg each) were mixed with 1.0 mL of extraction buffer (293.3 mmol/L sucrose, 246.4 mmol/L Tris-HCl, 69.4 mmol/L SDS, 0.51 mmol/L EDTA, 0.22 mmol/L Coomassie blue, 0.177 mmol/L phenol red, 0.105 mmol/L HCl, pH 8.5) containing DTT (50 mmol/L), incubated for 12 h, heated to 60 °C for 10 min and centrifuged (5000× *g*, 5 min, 20 °C). Ten µl of the supernatant were applied to the slots. The running buffer (MES buffer) consisted of 50 mmol/L 2-(*N*-morpholino)ethanesulfonic acid (MES), 50 mmol/L Tris-HCl, 3.5 mmol/L SDS, 1 mmol/L EDTA and 5 mmol/L DTT (pH 7.7). The running time was 30 min at 200 V and 115 mA. After the run, protein bands were fixed for 30 min in 12% (*w*/*w*) trichloroacetic acid, stained for 30 min with Coomassie blue and destained twice, first with methanol/acetic acid/water (50/10/40, *v*/*v*/*v*) and then with methanol/acetic acid/water (10/10/80, *v*/*v*/*v*). The gels were scanned, the images converted to grayscale, the lanes of interest plotted as x/y-diagrams and the peaks integrated using the Gel Doc^TM^ EZ gel documentation system (Bio-Rad Laboratories, Munich, Germany). 

### 2.9. Enzyme-Linked Immunosorbent Assay (ELISA)

The gluten content in the samples (gluten and pepgluten) was quantitated by a commercial competitive ELISA kit based on the R5 monoclonal antibody (RIDASCREEN^®^ Gliadin competitive, R-Biopharm, Darmstadt, Germany). The kit is intended to measure gluten contents in the 20 mg/kg and not in the 700 g/kg range, which is why the standard extraction procedure was modified. Gluten and pepgluten (10 mg, *n* = 3) were extracted with 2.5 mL Cocktail [31] at 50 °C for 40 min followed by the addition of 7.5 mL 80% (*v*/*v*) ethanol, shaking at 22 °C for 60 min and centrifugation (3750× *g*, 10 min, 22 °C). The resulting supernatant was subsequently diluted 1:100 and 1:50 with 60% (*v*/*v*) ethanol and finally 1:50 with the diluted sample buffer of the kit, resulting in a dilution factor of 2.5 × 10^7^. The following ELISA procedure was performed according to the kit manual in a separate, closed room where the surfaces had been cleaned with 60% ethanol (*v*/*v*) to prevent contamination with gluten. The absorbances were measured at 450 nm (Expert 96 microplate reader, Asys Hitech, Eugendorf, Austria) and the gluten concentrations calculated from the calibration curve using the cubic spline function of the RIDA^®^SOFT Win software (version z9996, R-Biopharm, Darmstadt, Germany).

### 2.10. Quantitation of ATIs

The contents of ATIs were determined based on five ATI-specific marker peptides by liquid chromatography-tandem mass spectrometry (LC-MS/MS) and stable isotope dilution assays (SIDA) with ^13^C- and ^15^N-labelled peptides as internal standards [32]. In brief, 10 mg of gluten and pepgluten (*n* = 3) were extracted twice with ammonium bicarbonate (0.5 mL, 50 mmol/L, pH 7.8). The combined extracts were lyophilized. After addition of the internal standards, the reconstituted ATI extract was reduced with tris(2-carboxyethyl)phosphine, alkylated with chloroacetamide, hydrolyzed with trypsin, lyophilized and re-dissolved in 1 mL of 0.1% (*v*/*v*) formic acid. Targeted LC-MS/MS was carried out with an UltiMate 3000 HPLC system (Dionex, Idstein, Germany) coupled to a triple-stage quadrupole mass spectrometer (TSQ Vantage, Thermo Fisher Scientific) exactly as described by Geisslitz et al. [32].

### 2.11. Descriptive Sensory Analysis

Ten healthy panelists (7 women and 3 men) with no history of known taste or smell disorders were trained in the sensory evaluation of aqueous solutions of the standard taste compounds sucralose (sweet), sodium chloride (salty), monosodium l-glutamate (umami), citric acid (sour), and caffeine (bitter) [33]. They gave written informed consent to participate in the sensory tests and had participated in sensory analyses for at least one year. The analyses were performed in a sensory panel room with separated booths at 20–22 °C. First, a descriptive analysis of the appearance, color and aroma of pepgluten was performed. Then, 2 g of pepgluten were mixed into 100 mL of different beverages with a small kitchen mixer. The beverages compared were mineral water, multivitamin juice, a mixture (70:30) of multivitamin juice and bitter lemon, grapefruit juice and different vegetable and fruit smoothies. The panel compared taste and aroma of the beverages with and without pepgluten.

### 2.12. Statistical Analysis

The software SigmaPlot 12.0 (Systat Software, San Jose, CA, USA) was used to assess significances of differences between the analytical parameters determined in both samples (gluten and pepgluten) with one-way analysis of variance (ANOVA) and Tukey’s test at *p* < 0.05. 

## 3. Results

### 3.1. Composition of Gluten

The qualitative and quantitative composition of gluten was determined to fulfill one of the most important requirements for oral food challenge materials. The nitrogen content of gluten was 126.1 mg/g, which corresponded to 718.8 mg/g crude protein using the conversion factor of 5.7 recommended for wheat proteins (ICC Standard Method 167). Further components in gluten were starch (107.7 mg/g), water (62.3 mg/g), dietary fiber (49.6 mg/g), fat (41.5 mg/g), and ash (7.9 mg/g), as seen in Table 1. The residue of 12.2 mg/g was very low (within the precision of the analytical methods used) and contained non-identified minor constituents that were not analyzed. The immunological determination of the gluten content using the R5 monoclonal antibody resulted in a gluten content of 646.6 mg/g, which corresponds to 90% of crude protein. Targeted LC-MS/MS SIDA based on five ATI-specific marker peptides revealed that 32.6 mg/g total ATIs were present in gluten, with CM3 (11.9 mg/g) and 0.19 + 0.53 (10.5 mg/g) as predominant ATI types. 

### 3.2. Composition of the Protein Fraction in Gluten

**Figure 1:**
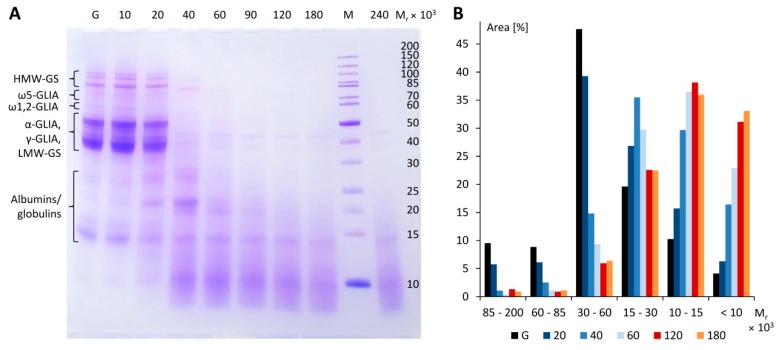
Sodium dodecyl sulfate-polyacrylamide gel electrophoresis (SDS-PAGE) of gluten (G) and different peptic digests incubated at 37 °C for 10 min, 20 min, 40 min, 60 min, 90 min, 120 min, 180 min and 240 min at an E:G:S ratio of 1:25:100 (**A**). Area (%) relative to the total area of selected bands according to molecular weight ranges of marker M (**B**). HMW-GS, high-molecular-weight glutenin subunits, LMW-GS, low-molecular-weight glutenin subunits, M, molecular weight marker, M_r_, relative molecular weight, α-GLIA, α-gliadins, γ-GLIA, γ-gliadins, ω5-GLIA, ω5-gliadins, ω1,2-GLIA, ω1,2-gliadins.

SDS-PAGE of gluten, seen in Figure 1A, lane G, showed the typical protein bands known from wheat flours including the following gluten protein types and ranges of M_r_ (according to known amino acid sequences): HMW-GS (67–88 × 10^3^), ω5-GLIA (49–55 × 10^3^), ω1,2-GLIA (39–44 × 10^3^), LMW-GS, α-GLIA and γ-GLIA (28–39 × 10^3^) and ALGL (10–25 × 10^3^).

**Figure 2:**
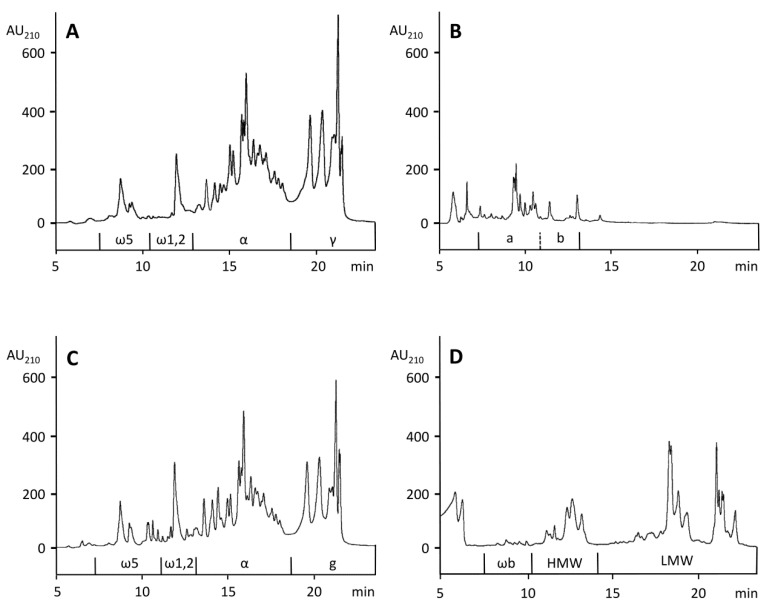
Reversed-phase high-performance liquid chromatography (RP-HPLC) for gluten proteins of prolamin working group (PWG)-gliadin (**A**), the ALGL fraction (**B**), the ALGL + gliadins (GLIA) fraction (**C**) and the glutenin subunits (GLUT) fraction (**D**). AU_210_, absorbance units at 210 nm, ALGL, albumins and globulins, HMW, high-molecular-weight glutenin subunits, LMW, low-molecular-weight glutenin subunits, α, α-gliadins, γ, γ-gliadins, ω5, ω5-gliadins, ω1,2, ω1,2-gliadins, ωb, ωb-gliadins.

The contents of ALGL, GLIA, and GLUT present in gluten were determined according to a combined extraction/RP-HPLC method developed for the analysis of wheat flour [26]. An advantage of RP-HPLC is that each gliadin and glutenin protein type is separated largely as a subgroup and can be quantitated without major overlap. As an example, the elution regions of ω5-, ω1,2, α-, and γ-GLIA are shown in Figure 2A for PWG-gliadin, which was used for calibration. However, the method had to be modified, because, in contrast to flour, gluten proteins strongly agglomerated after the initial extraction of ALGL with salt solution. Therefore, the following extraction of GLIA with 60% ethanol was not reproducible (coefficient of variation > 20%) and the procedure was divided into two separate experiments: First, ALGL were extracted from gluten with salt solution using three extraction steps. Second, gluten was extracted with 60% ethanol using three extraction steps to obtain a mixture of ALGL and GLIA. Then, GLUT were extracted three times with glutenin extraction solvent from the residue of ALGL + GLIA. Single corresponding extracts were combined into the three fractions ALGL, ALGL + GLIA and GLUT and analyzed by RP-HPLC, as seen in Figure 2B–D.

For the quantitation of GLIA, the value of ALGL had to be subtracted from the value of the ALGL + GLIA fraction. GLIA were predominant (402.1 mg/g), followed by GLUT (213.8 mg/g) and ALGL (27.7 mg/g), as seen in Table 2. The gluten content, as sum of GLIA and GLUT, amounted to 615.9 mg/g and the ratio of GLIA to GLUT was 1.88. The contents of gluten protein types were also calculated from the respective areas, again using the ALGL + GLIA chromatogram minus the ALGL chromatogram. The ω5-GLIA and ω1,2-GLIA were most affected by co-eluting ALGL, as 72.5% of ALGL were eluted in the region of ω5-GLIA and 27.5% in the region of ω1,2-GLIA. Corresponding proportions of ALGL, shown in Figure 2B, areas a and b, were subtracted from ω5- and ω1,2-GLIA, respectively, as seen in Figure 2C. The elution profiles of α- and γ-GLIA from PWG-gliadin isolated from a mixture of 28 wheat cultivars [23] and of gluten (mixture of unknown cultivars), seen in Figure 2A,C, were almost identical. In total, α-GLIA (208.8 mg/g), γ-GLIA (149.2 mg/g) and LMW-GS (149.1 mg/g) belonged to the major protein types, while ω5-GLIA (16.3 mg/g), ω1,2-GLIA (27.8 mg/g), ωb-GLIA (9.7 mg/g) and HMW-GS (55.0 mg/g) were minor components, as shown in Table 2.

### 3.3. Optimization of Gluten Hydrolysis

The first experiment was performed to test the activity of the commercial pepsin dietary supplement. Each capsule (203 mg weight on average) contained 153 mg pepsin (manufacturer’s information). The analysis of nitrogen (177.7 mg/g) indicated that the capsules exclusively consisted of protein (101.3 ± 0.1%, *n* = 3), including the main component pepsin (≈75%) and also gelatin for pill preparation. Gluten was first incubated for 120 min at 37 °C at an E:G:S ratio of 1:25:1667 [28] using hydrochloric acid (0.01 mol/L, pH 2.0). The analysis of aliquots taken during the whole digestion period by RP-HPLC (for the gluten hydrolysates) indicated that gluten proteins were extensively degraded with an endpoint at 120 min. After that time, there was no further increase in absorbance areas.

Having thus confirmed the activity of the pepsin capsules, hydrochloric acid was replaced by food-grade acetic acid (acetic acid essence mixed with mineral water to obtain pH 3.0) and the solvent volume was decreased (E:G:S ratios of 1:25:200, 1:25:150 and 1:25:100) to speed up the following drying process. RP-HPLC and SDS-PAGE analyses revealed that the progress of gluten degradation was only marginally different compared to the E:G:S ratio of 1:25:1667. SDS-PAGE, shown in Figure 1A, confirmed that gluten proteins (M_r_ = 30–100 × 10^3^ in a reduced state, according to the marker) were successively degraded during incubation and that the hydrolysis reached an endpoint at 120 min, because there was no further significant change compared to 180 min, as seen in Figure 1B. While gluten had an approximate distribution of 9% of proteins with M_r_ = 85–200 × 10^3^ (HMW-GS), 9% (M_r_ = 60–85 × 10^3^, ω5- and ω1,2-gliadins), 48% (M_r_ = 30–60 × 10^3^, α- and γ-gliadins and LMW-GS), 20% (M_r_ = 15–30 × 10^3^), 10% (M_r_ = 10–15 × 10^3^) and 4% (M_r_ < 10 × 10^3^), this distribution fundamentally changed to 1%, 1%, 6%, 23%, 38% and 31%, respectively, after an incubation time of 120 min. The gel thus confirmed extensive degradation of gluten proteins and revealed that about 70% of the hydrolysate was composed of gluten fragments with M_r_ of 15 × 10^3^ and below.

**Figure 3:**
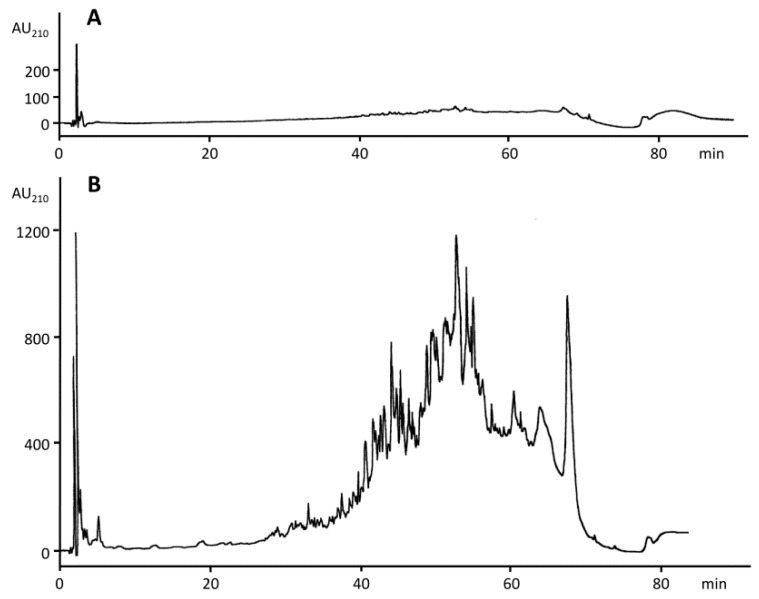
RP-HPLC (peptide system) of the peptic hydrolysis of gluten after incubation at 37 °C for 0 min (**A**) and 120 min (**B**). The ratio of enzyme to gluten to solvent was 1:25:100. AU_210_, absorbance units at 210 nm.

RP-HPLC revealed that gluten did not contain any fragments, as seen in Figure 3A, whereas the gluten hydrolysate showed a complex fragment mixture after 120 min, see in Figure 3B. 

To test whether the amount of pepsin could be reduced, the following E:G:S ratios were tested: 1:50:200, 1:100:400 and 1:200:800. SDS-PAGE after an incubation time of 120 min each (not shown) indicated that fragments with high M_r_ were generally more dominant than in the digests with E:G:S = 1:25:100. Moreover, the lyophilized material suspended in mineral water remained glutinous. Altogether, the following optimized procedure for gluten hydrolysis was used for further experiments: E:G:S ratio of 1:25:100 with food-grade acetic acid (pH 3.0) as solvent and an incubation at 37 °C for 120 min.

### 3.4. Preparation and Characterization of Pepgluten 

The final gluten hydrolysate (called “pepgluten”) was prepared from 95 g gluten digested with 3.8 g pepsin (25 capsules weighing 5 g) in 380 mL acetic acid (pH 3.0) at 37 °C for 120 min (E:G:S = 1:25:100). After freezing, the preparation was lyophilized. The inactivation of the enzyme by heating was not performed in acetic acid to avoid partial deamidation of glutamine side chains, which might influence the immunoreactivity of pepgluten. Therefore, pepsin was inactivated by suspending the lyophilized digest in boiling mineral water for 10 min. An additional reason for this procedure was that heating created a more realistic material for gluten challenge tests, because most gluten-containing foods such as bread or pasta are also baked or cooked before consumption. After the second lyophilization, the dry material was milled and stored in a desiccator over sodium hydroxide (baker’s brine) until constant weight. Pepgluten was not separated into soluble and insoluble parts, as had been done previously by Frazer et al. [34] (FIII and FVI) and others, who subsequently studied only the soluble part. Thus, the proportions of gluten proteins remained unchanged, which was important for the calculation of gluten equivalents in the hydrolysate.

The yield of dried pepgluten (92.6 g) was 92.2%, based on the amounts of non-volatile substances used for the digestion (95 g vital gluten, 5.1 g pepsin capsules and 0.3 g salts in the mineral water). Considering the frequent transfers of solutions and substances during the processes of digestion, freeze-drying, heating, milling and storage, the obtained yield appeared to be satisfactory. Compared to gluten as starting material, there were no significant differences between both materials for contents of protein (707.1 mg/g), starch (104.2 mg/g), fat (46.6 mg/g), dietary fiber (40.5 mg/g) and only slight differences in water (59.2 mg/g) and ash (10.9 mg/g) contents due to drying and addition of salts from the mineral water, as seen in Table 1. The gluten content of the hydrolysate (372 mg/g) determined by the R5 competitive ELISA, shown in Table 1, was reduced compared to gluten. The contents of ATIs 0.19 + 0.53, 0.28, CM2 and CM16 were not significantly different, but the content of CM3 was lower (2.7 mg/g) in pepgluten compared to the starting material (11.9 mg/g).

Finally, the contents of gluten equivalents in the hydrolysate were calculated based on its crude protein content (707 mg/g = 100%) considering the proportions of gluten proteins and pepsin capsule proteins in the digestion assay, as shown in Table 2. Accordingly, 1 g of pepgluten contained gluten equivalents derived from 369 mg GLIA and 196 mg GLUT resulting in 565 mg total gluten equivalents. Further protein components of pepgluten were derived from ALGL (25 mg/g), residual proteins (69 mg/g) and pepsin capsule proteins (48 mg/g), as seen in Table 2.

### 3.5. Descriptive Sensory Analysis

The pepgluten powder was slightly brownish in appearance and had a flour- and sourdough-like aroma with a residual slightly pungent note originating from acetic acid. When mixed into mineral water (2 g in 100 mL), the drink was turbid and had a dough-like aroma and bitter taste. The intensity of aroma and taste was less noticeable in a turbid multivitamin juice, which appeared to be suitable for an open challenge test. If necessary, the endogenous bitter taste can be completely covered by mixing multivitamin juice with bitter lemon (70:30 *v*/*v*) or by using grapefruit juice. Different vegetable or fruit smoothies also allowed the complete camouflage of the preparation.

## 4. Discussion

Due to its glutinous properties, wheat gluten is difficult to handle for oral food challenge and in cell-based assays for clinical investigations, because these only tolerate aqueous solvents. Therefore, gluten is commonly applied in form of the soluble portions of peptic [34,35], tryptic [35], chymotryptic [5], peptic-tryptic [8,36,37], peptic-chymotryptic [37] or peptic-tryptic-chymotryptic [28] hydrolysates, sometimes in combination with further enzymes such as elastase or carboxypeptidase to mimic human gastrointestinal digestion as closely as possible. Gluten contains very low amounts of arginine and lysine (preferential cleavage sites for trypsin) and cleavage after aromatic amino acid residues phenylalanine, tryptophan, and tyrosine (preferential cleavage sites for chymotrypsin) is often impeded by a following proline residue [6]. Pepsin cleaves before or after phenylalanine, tryptophan, tyrosine, and leucine and has the broadest repertoire of cleavage sites of the three enzymes, especially considering the content of repetitive sequences rich in proline and glutamine that are typical of gluten proteins [38]. Therefore, pepsin was selected to prepare pepgluten. For patient safety [11,12], the procedure was carried out in a kitchen using only food-grade ingredients: gluten (baking ingredient), pepsin (dietary supplement), acetic acid essence (for meals and salads) and mineral water in a glass bottle. 

The composition of gluten proteins in the commercial gluten used as a starting material was similar to that expected from wheat flours [7]. Due to the stretched conformation of reduced gluten proteins, their SDS-PAGE mobility is known to be restricted compared to the globular proteins used in the marker, so that, e.g., HMW-GS (M_r_: 67–88 × 10^3^ based on known amino acid sequences) appeared between M_r_: 85–120 × 10^3^, as has been reported before [5,30]. The contents, elution profiles of GLIA and GLUT and their ratio (1.88) were very similar to those reported for common wheat flour [39]. In contrast, ALGL, usually present in wheat flours with contents of 150–200 mg/g, were almost absent due to the washing steps during gluten-starch separation. Judging by the overall gluten protein composition, the gluten sample appeared to be representative. The most important conclusion of the analytical characterization was, however, that commercial gluten contains a much lower amount of gluten proteins (≈62%) than is typically assumed (100%). This implies that the true amount of gluten applied would have to be reconsidered in many cases.

Pepgluten showed no significant differences to gluten in the contents of protein, starch, fat, dietary fiber, and ATIs 0.19 + 0.53, 0.28, CM2 and CM16 and only slight differences in water and ash contents due to drying and addition of mineral water. Only CM3 appeared to be susceptible to peptic hydrolysis, because the content of the marker peptide decreased to about 23%. The immunological quantitation of gluten in the hydrolysate by R5 competitive ELISA only resulted in about 58% recovery compared to the starting material. This reduced recovery after enzymatic digestion is a well-known general effect regardless of type of ELISA and antibody and has been reported for peptic-tryptic gliadin (63% recovery) [37], peptic wheat flour gluten proteins (47% recovery) [35], peptic-tryptic gliadin (56% recovery), and peptic-tryptic glutenin (23% recovery) [36]. It is uncertain whether ELISA, even in a competitive format, is the most robust assay to determine partially hydrolyzed gluten [36], which is why the calculation of gluten equivalents in the hydrolysate was performed based on results for crude protein and RP-HPLC. Further insight into the composition of pepgluten may be gained using untargeted proteomics approaches. 

Pepgluten has many potential applications, e.g., as material for oral food challenge for CD patients who started a gluten-free diet prior to appropriate diagnosis [10], for wheat allergy [11] and WDEIA patients [13,40], and for NCGS and IBS patients [2,3]. It is also suitable for in vitro testing on, e.g., human monocytes [8] or for studies on small intestinal permeability with subsequent detection of gluten fragments in urine samples [41]. The commonly used lactulose/mannitol (LAMA) test [42] to assess permeability (“leaky gut”) does not appear to be well suited as an indicator for the intestinal passage of larger molecules [10], so that an oral food challenge with pepgluten and detection of gluten fragments in blood, urine, or feces might be more meaningful.

## 5. Conclusions

Commercially available wheat gluten was extensively characterized and used as a starting material to prepare a well-defined, food-grade wheat gluten hydrolysate (pepgluten). Since no material was removed during the procedure, the peptic hydrolysate had a composition very similar to the original gluten and contained all components relevant for wheat hypersensitivities including ATIs. Based on food-grade ingredients only, it is deemed to be suitable for oral food challenge tests. Pepgluten fulfills important criteria for challenge materials, because its composition is known, it is easy to apply and, mixed with suitable beverages, it is indistinguishable from placebo samples regarding appearance, smell, taste and texture. Altogether, pepgluten is appropriate as standard preparation for oral provocation tests and clinical investigations in the field of wheat hypersensitivities.

## Figures and Tables

**Table 1:** Composition of gluten and pepgluten. ATIs: α-amylase/trypsin-inhibitors.

Constituent	Gluten	Pepgluten
	(mg/g) ^1^	(mg/g) ^1^
Crude protein (nitrogen × 5.7)	718.8 ± 1.8 ^A^	707.1 ± 2.5 ^A^
Starch	107.7 ± 3.5 ^A^	104.2 ± 1.0 ^A^
Water	62.3 ± 0.3 ^A^	59.2 ± 1.9 ^B^
Fat	41.5 ± 2.7 ^A^	46.6 ± 0.7 ^A^
Dietary fiber	49.6 ± 4.7 ^A^	40.5 ± 4.3 ^A^
Ash	7.9 ± 0.1 ^A^	10.9 ± 0.1 ^B^
Residue	12.2	31.5
Gluten ^2^	646.6 ± 34.8 ^A^	372.0 ± 36.3 ^B^
ATIs (sum)	32.6 ^A^	22.4 ^B^
ATI 0.19 + 0.53	10.5 ± 1.6 ^A^	10.6 ± 1.4 ^A^
ATI 0.28	2.0 ± 0.2 ^A^	1.9 ± 0.1 ^A^
ATI CM2	1.7 ± 0.3 ^A^	1.6 ± 0.1 ^A^
ATI CM3	11.9 ± 0.4 ^A^	2.7 ± 0.1 ^B^
ATI CM16	6.5 ± 1.1 ^A^	5.6 ± 0.9 ^A^

^1^ Results are given as mean ± standard deviation of triplicate determinations and different superscript letters designate significant differences between the two samples (one-way analysis of variance (ANOVA), Tukey’s test, *p* < 0.05, ^2^ Gluten content determined by R5 competitive enzyme-linked immunosorbent assay (ELISA).

**Table 2:** Composition of the protein fraction in gluten and pepgluten.

Protein	Gluten	Gluten Digestion Assay		Pepgluten
	(mg/g) ^1^	(mg/g) ^2^	(%)	(mg/g)
ALGL	27.7 ± 0.8	26.3	3.6	25.4
GLIA	402.1 ± 5.4	382.0	52.1	368.6
ω5	16.3 ± 1.1	15.5	2.1	15.0
ω1,2	27.8 ± 0.6	26.4	3.6	25.5
α	208.8 ± 2.9	198.4	27.1	191.4
γ	149.2 ± 1.2	141.7	19.3	136.7
GLUT	213.8 ± 6.1	203.1	27.7	196.0
ωb	9.7 ± 0.5	9.2	1.3	8.9
HMW-GS	55.0 ± 1.5	52.3	7.1	50.5
LMW-GS	149.1 ± 3.9	141.6	19.3	136.6
Gluten ^3^	615.9 ± 2.9	585.1	79.8	564.6
Residue	75.2	71.4	9.8	68.9
Capsule ^4^	-	50.0	6.8	48.2
Sum	718.8 ± 1.8	732.8	100	707.1 ± 2.5

^1^ Results are given as mean ± standard deviation (*n* = 3), ^2^ calculated from 95 g gluten and 5 g pepsin capsule, ^3^ sum of GLIA and GLUT, ^4^ protein content in the pepsin capsule, ALGL, albumins and globulins, HMW, high-molecular-weight glutenin subunits, LMW, low-molecular-weight glutenin subunits, α, α-gliadins, γ, γ-gliadins, ω5, ω5-gliadins, ω1,2, ω1,2-gliadins, ωb, ωb-gliadins.
